# Supplementary material for: Drug enrichment and discovery from schizophrenia genome-wide association results: an analysis and visualisation approach
Source: Sci Rep. 2017 Sep 29;7:12460. doi: 10.1038/s41598-017-12325-3 (PMC5622077; doi:10.1038/s41598-017-12325-3)
Supplement: Supplementary file 1 — Supplement 1 [file 41598_2017_12325_MOESM1_ESM.pdf]

Supplement 1 - Drug enrichment and discovery from schizophrenia genome-wide association results: an analysis and visualisation approach

H. A. Gaspar[1,2,]* and G. Breen[1,2]

Supplementary Figures

Figure S1. Association between druggable genes and schizophrenia in SCZ-PGC2. (**a**) Gene Manhattan plot for 4298 druggable genes, showing the schizophrenia SCZ-PGC2 association (-$\log_{10}$(p-value)) as a function of chromosomal position. The gene with the lowest p-value for each chromosome was annotated. The red line indicates the Bonferroni threshold at $\alpha = 5\%$. (**b**) Significant druggable genes (Bonferroni threshold) belonging to the same gene families, with at least 3 significant genes.

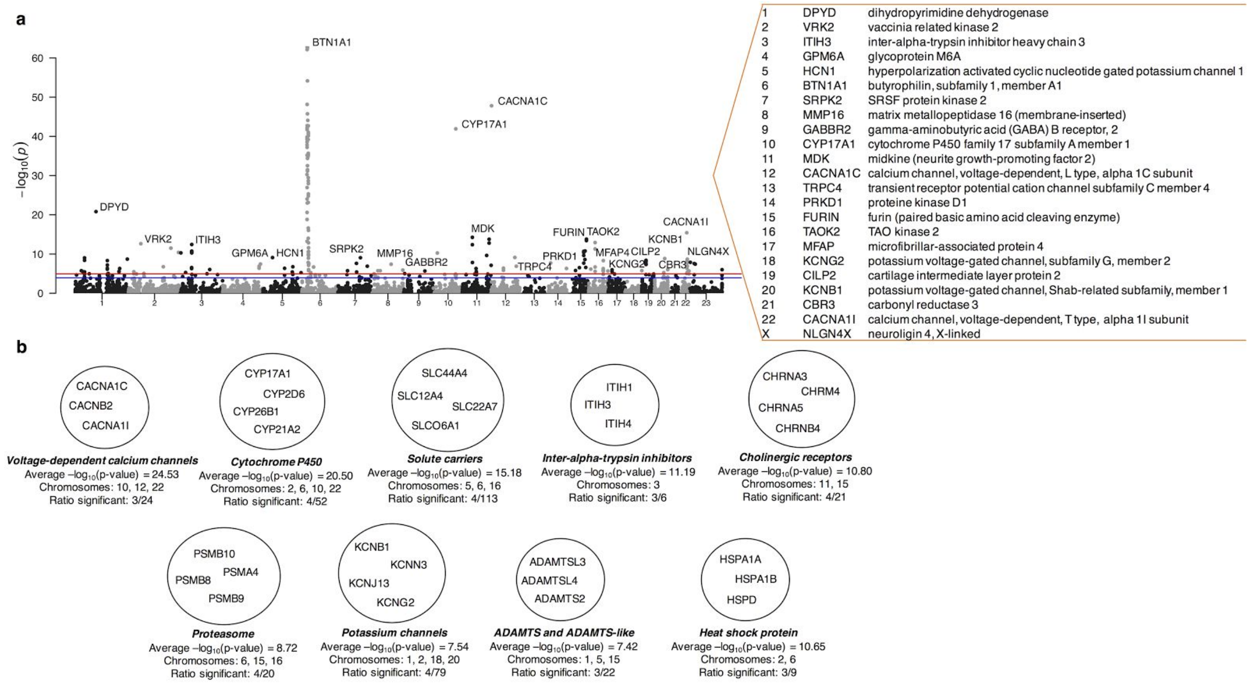

Figure S2. Association (-$\log_{10}$(p-value)) of voltage dependent calcium channels and neurotransmitter receptors with schizophrenia (SCZ-PGC2), with Bonferroni significance threshold: (**a**) voltage-dependent calcium channels, (**b**) nicotinic receptors, (**c**) dopamine receptors, (**d**) serotonin receptors, (**e**) GABA receptors, (**f**) glutamate receptors, (**g**) epinephrine receptors, and (**e**) opioid and somatostatin receptors.

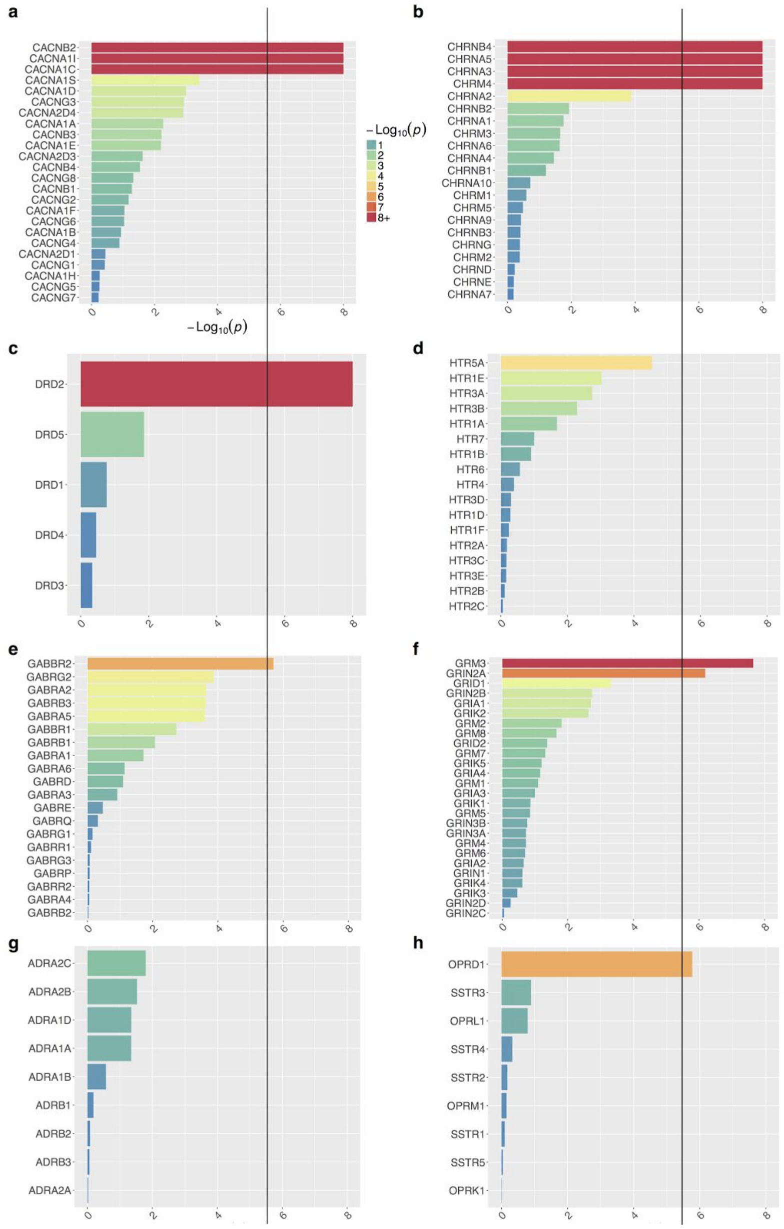

Figure S3. Region LocusZoom plots[1] of (**a**) *OPRD1*, (**b**) *GABBR2*, and (**c**) *NOS1* with +/- 500Kb windows (NBI build 37). The top SNP is indicated in purple and the colour of all other SNPs is representative of the pairwise r^2 using LD data from 1000 Genomes EUR.

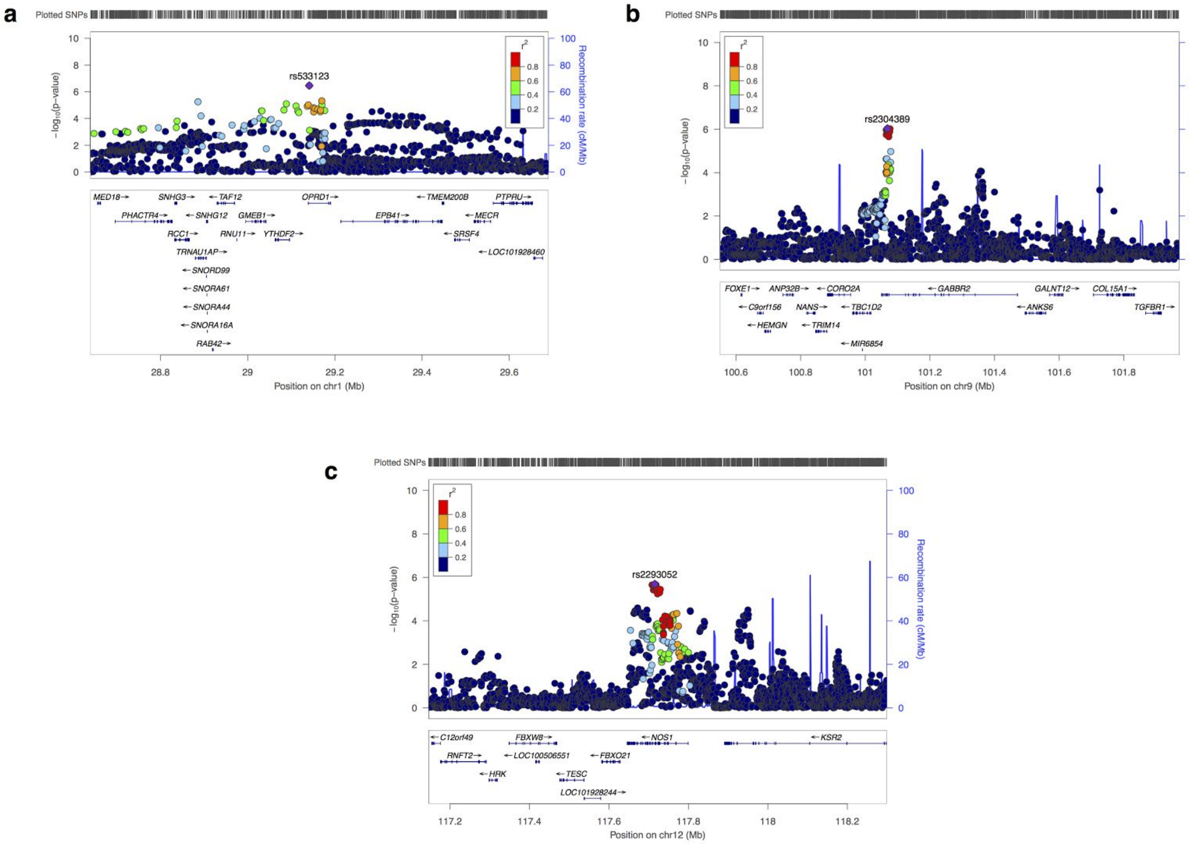

1. Pruim, R. J. *et al.* LocusZoom: regional visualization of genome-wide association scan results. *Bioinformatics* **26,** 2336–2337 (2010).

Figure S4. STRINGdb[1] protein-protein interaction (PPI) network of 123 druggable genes significant in SCZ-PGC2.

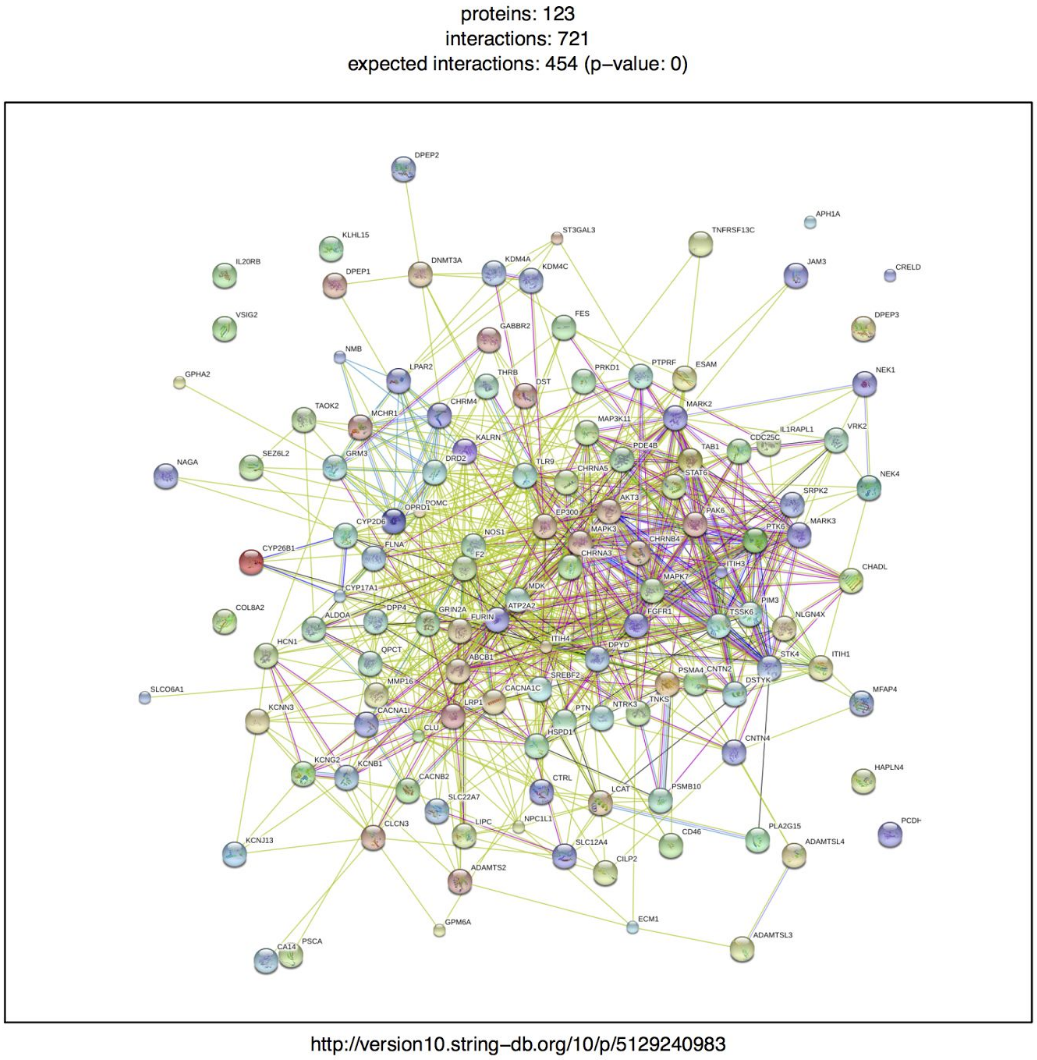

1. Szklarczyk, D. *et al.* STRING v10: protein-protein interaction networks, integrated over the tree of life. *Nucleic Acids Res.* **43,** D447–52 (2015).

Supplementary Tables

Table S1. Three schizophrenia GWASs with different sample sizes. The number of cases and controls as well as the genomic inflation factor (λ_{GC}) and LD score intercept were computed for each study.

GWAS	λ_{GC}	*ldscore intercept*	*Cases*	*Controls*	*Cases/Controls*
SCZ-PGC1	1.23	1.02	9,394	12,462	0.75
SCZ-PGC1+SWE	1.30	1.01	13,833	18,310	0.76
SCZ-PGC2	1.58	1.04	35,476	46,839	0.76

Table S2. Drugs prescribed for schizophrenia in *The Maudsley Prescribing Guidelines in Psychiatry*. The table indicates the ATC (Anatomical Therapeutic Chemical) code, class, and the generation (1 = first generation, 2 = second generation) of antipsychotics.

Maudsley SCZ	*ATC code*	*ATC class*	*Type*
chlorpromazine	N05AA01	Phenothiazines with aliphatic side-chain	1
levopromazine	N05AA02	Phenothiazines with aliphatic side-chain	1
fluphenazine	N05AB02	Phenothiazines with piperazine structure	1
perphenazine	N05AB03	Phenothiazines with piperazine structure	1
trifluoperazine	N05AB06	Phenothiazines with piperazine structure	1
periciazine	N05AC01	Phenothiazines with piperidine structure	1
pipotiazine	N05AC04	Phenothiazines with piperidine structure	1
haloperidol	N05AD01	Butyrophenone derivatives	1
flupentixol	N05AF01	Thioxanthene derivatives	1
zuclopenthixol	N05AF05	Thioxanthene derivatives	1
pimozide	N05AG02	Diphenylbutylpiperidine derivatives	1
loxapine	N05AH01	Diazepines, oxazepines, thiazepines, oxepines	1
sertindole	N05AE03	Indole derivatives drugs	2
ziprasidone	N05AE04	Indole derivatives drugs	2
clozapine	N05AH02	Diazepines, oxazepines, thiazepines, oxepines	2
olanzapine	N05AH03	Diazepines, oxazepines, thiazepines, oxepines	2
quetiapine	N05AH04	Diazepines, oxazepines, thiazepines, oxepines	2
asenapine	N05AH05	Diazepines, oxazepines, thiazepines, oxepines	2
sulpiride	N05AL01	Benzamide antipsychotics	2
amisulpride	N05AL05	Benzamide antipsychotics	2
risperidone	N05AX08	Other antipsychotics	2
aripiprazole	N05AX12	Other antipsychotics	2
paliperidone	N05AX13	Other antipsychotics	2
iloperidone	N05AX14	Other antipsychotics	2

Table S3. Top 10 druggable gene families in schizophrenia GWAS SCZ-PGC2. The p-value from the competitive test is provided as well as the Benjamini and Hochberg FDR-adjusted p-value (q-value). The gene families were defined using the HUGO nomenclature.

Gene Family	*N*	*p-value*	*q-value*
Cytochrome P450 family	2	2.23×10^{-7}	1.84×10^{-4}
Calcium voltage-gated channel subunits	26	5.40×10^{-6}	2.53×10^{-3}
Killer cell immunoglobulin like receptors	9	5.07×10^{-4}	5.42×10^{-2}
ZF class homeoboxes and pseudogenes	15	5.68×10^{-3}	0.204
Sulfotransferases, cytosolic	14	1.01×10^{-2}	0.263
Protein phosphatase 1 regulatory subunits	177	1.26×10^{-2}	0.289
Butyrophilins	14	1.44×10^{-2}	0.305
EF-hand domain containing	215	1.61×10^{-2}	0.318
Kruppel like factors	17	1.69×10^{-2}	0.326
Histocompatibility complex	35	2.16×10^{-2}	0.361

Table S4. Top 10 Open Targets diseases and phenotypes in GWAS SCZ-PGC2. The p-value from the competitive test is provided as well as the Benjamini and Hochberg FDR-adjusted p-value (q-value). *EPILEPSY INTERSECT* gathers genes shared among Open Targets epilepsy pathways.

Pathway	*N*	*p-value*	*q-value*
SCHIZOPHRENIA	2579	3.24×10^{-47}	2.20×10^{-43}
MENTAL OR BEHAVIOURAL DISORDER	5698	1.70×10^{-14}	5.77×10^{-11}
GENETIC CARDIAC ANOMALY	1263	1.70×10^{-6}	1.05×10^{-3}
CLASSIC CONGENITAL ADRENAL HYPERPLASIA DUE TO 21-HYDROXYLASE DEFICIENCY	17	1.92×10^{-6}	1.13×10^{-3}
INTERAURICULAR COMMUNICATION	988	3.46×10^{-6}	1.81×10^{-3}
EPILEPSY SYNDROME	801	1.51×10^{-5}	5.69×10^{-3}
REFLEX SYMPATHETIC DYSTROPHY	45	2.12×10^{-5}	7.02×10^{-3}
GENICULATE HERPES ZOSTER	30	3.48×10^{-5}	1.05×10^{-2}
NEONATAL EPILEPSY SYNDROME	203	3.71×10^{-5}	1.09×10^{-2}
EPILEPSY INTERSECT	2922	3.76×10^{-5}	1.09×10^{-2}

Table S5. Top 10 GO pathways in GWAS SCZ-PGC2. The p-value from the competitive test is provided as well as the FDR-adjusted p-value (q-value).

Pathway	*N*	*p-value*	*q-value*
GO: GLUCOCORTICOID BIOSYNTHETIC PROCESS	11	4.00×10^{-8}	5.74×10^{-5}
GO: NEURON PROJECTION	920	6.68×10^{-8}	7.56×10^{-5}
GO: REGULATION OF SYNAPTIC PLASTICITY	137	9.85×10^{-8}	1.03×10^{-4}
GO: VOLTAGE GATED CALCIUM CHANNEL COMPLEX	39	1.50×10^{-7}	1.45×10^{-4}
GO: CALCIUM CHANNEL COMPLEX	59	2.39×10^{-7}	1.84×10^{-4}
GO: GLUCOCORTICOID METABOLIC PROCESS	16	2.44×10^{-7}	1.84×10^{-4}
GO: DNA REPLICATION DEPENDENT NUCLEOSOME ASSEMBLY	32	3.26×10^{-7}	2.33×10^{-4}
GO: T TUBULE	45	1.50×10^{-6}	9.69×10^{-4}
GO: PROTEIN HETEROTETRAMERIZATION	38	2.84×10^{-6}	1.61×10^{-3}
GO: MEMBRANE DEPOLARIZATION DURING ACTION POTENTIAL	39	4.04×10^{-6}	1.96×10^{-3}

Text S1: *Drug gene-sets from K_i DB and DGIdb .*

Drug gene-sets were extracted from K_i DB and DGIdb drug/gene interaction databases. We applied several filters listed in Tables 1-2, and merged the two databases (cf. Table 3).

Table 1. K_i DB filtering.

With non-empty K_i field	59,646
Only Human	32,831
K_i not superior or inferior to a value	24,011
With molecule name	23,447
With gene name	18,019
Unique pairs	12,540
With range(pK_i)<2	12,424
Final interactions with midrange pK_i >= 5 and < 14	11,822
Number of gene-sets	4,461
Number of unique gene-sets	606
Number of unique gene-sets of size ≥ 2	510
Degenerescence (molecules/gene-set)	7.36

Table 2. DGIdb filtering.

Number of interactions	32,108
Number of gene-sets	10,922
Number of unique gene-sets	3,622
Number of unique gene-sets of size ≥ 2	2,423
Degenerescence (molecules/gene-set)	3.02

Table 3. Merging K_i DB and DGIdb.

Number of interactions	35,098
Number of gene-sets	14,917
Number of unique gene-sets	3,939
Number of unique gene-sets of size ≥ 2	2,737
Degenerescence (molecules/gene-set)	3.79

Text S2: *Pathway analysis in MAGMA.*

The gene association vector **Z** with elements $Z_{gene\,1}$, $Z_{gene\,2} \ldots Z_{gene\,n}$ can be used in a regression model for each pathway p

$$Z = \alpha_p \vec{1} + \beta_{1p} x_{1p} + \beta_2 x_2 + \beta_3 x_3 + \ldots + \varepsilon$$

They are two types of pathway analysis: self-contained and competitive. The self-contained analysis tests whether a pathway is associated or not with the trait; the competitive analysis tests whether genes in the pathway are more associated than other genes. If all parameters β are equal to 0, and if only Z values within pathway p are taken into account (Z_p), the model is intercept-only and a p-value can be obtained by testing $\alpha_p > 0$ against the null hypothesis $\alpha_p = 0$. This is the self-contained p-value, testing whether the mean gene association value α_p within the pathway is significantly above 0. If, instead, all parameters β are not equal to 0 (competitive analysis), β_{1p} reflects the difference between the gene associations within and outside the pathway, and x_{1p} is a binary vector with ith element = 1 if the ith gene is within pathway p and = 0 otherwise. The competitive p-value is obtained by testing $\beta_{1p} > 0$ (better association within the pathway) against the null hypothesis $\beta_{1p} = 0$ (no difference in association within or outside the pathway).

In MAGMA, other variables (x_2, $x_3 \ldots$) are used to account for gene size, gene density, minor allele count, and the log of those values. The gene density is the ratio of gene size to the number of SNPs in the gene. Because of the LD between genes the errors may be correlated, therefore a generalized least squares approach is adopted where residuals have the variance $\sigma^2\Sigma$, where Σ is the gene correlation matrix.

Text S3: *Hub genes in PPI network.*

The 123 significant druggable genes were used to build two STRINGdb protein-protein interaction subnetworks. One subnetwork, *d*, was only constructed with the 123 genes (cf. **Supplementary Figure S4**); the other, *g*, was constructed with the 123 genes as well as all protein-coding genes significant in schizophrenia, for a total of 498 genes. We used normalized node betweenness and node degree measure to find hub genes, averaged between values drawn from *d* and *g* subnetworks. The betweenness, normalized betweenness, and degree of each node in the network was computed using the *igraph* R package.

Betweenness *b* of a node (gene) is the sum of the fraction of shortest paths that pass through the node. The normalized betweenness is computed as $b' = 2*b/(n*n-3*n+2)$, where *n* is the number of nodes.

A normalized degree measure was computed for each node in the network, by dividing the degree n of a node in the subnetwork by the degree in the complete network. The normalized degree measure in the significant druggable subnetwork *d* is obtained by dividing by the degree in the whole druggable network *D*, and the value for the significant protein-coding subnetwork *g* is obtained by dividing by the degree in the whole protein-coding network *G*.
